# Is Gestational Diabetes Mellitus a Risk Factor of Maternal Breast Cancer? A Systematic Review of the Literature

**DOI:** 10.3390/biomedicines9091174

**Published:** 2021-09-07

**Authors:** Julien Simon, Karine Goueslard, Sonia Bechraoui-Quantin, Patrick Arveux, Catherine Quantin

**Affiliations:** 1 Biostatistics and Bioinformatics (DIM), University Hospital, 21000 Dijon, France; simonjulien@free.fr (J.S.); karine.goueslard@chu-dijon.fr (K.G.); s.bechraouiquantin@student.helmo.be (S.B.-Q.); 2 Center for Primary Care and Public Health, Unisanté, University of Lausanne, 1010 Lausanne, Switzerland; Patrick.Arveux@unisante.ch; 3 Inserm, CIC 1432, Clinical Investigation Center, Clinical Epidemiology/Clinical Trials Unit, Dijon University Hospital, 21000 Dijon, France; 4 High-Dimensional Biostatistics for Drug Safety and Genomics, Université Paris-Saclay, UVSQ, University Paris-Sud, Inserm, CESP, 94800 Villejuif, France

**Keywords:** systematic review, gestational diabetes mellitus, breast cancer, risk factor, qualitative analysis

## Abstract

The association between gestational diabetes mellitus (GDM) and breast cancer (BC) risk is complex. We aimed to examine this association in a systematic review of the literature. This review was done using the PubMed/Medline and Web of Science databases, in accordance with the Preferred Reporting Items for Systematic Reviews and Meta-Analyses (PRISMA) guidelines. The Newcastle–Ottawa Scale was used for the assessment of bias and quality of studies. Only English-language articles published before 1 June 2021, were included. Fourteen studies were included in this systematic review. Among them, eight did not find statistically significant results. Three studies showed a statistically significant increased risk of BC after GDM, and they explained this potential increased risk by hyperinsulinemia, hyperglycemia, and low-grade inflammation. However, three studies showed a statistically significant decreased risk of BC after GDM, suggesting a possible protective effect of hormonal changes induced by GDM during pregnancy. These controversial results should be interpreted with caution due to both quantitative and qualitative methodological shortcomings. Further investigations are thus needed in order to gain a better understanding of the associations between GDM and BC, and their underlying mechanisms.

## 1. Introduction

Breast cancer (BC) is the most common cancer, accounting for about one fourth of all cancer cases in women in Europe and worldwide [1].

Women with diabetes have a 15–20% increased risk of BC compared to women without diabetes [2,3,4,5,6,7,8]. Underlying mechanisms include the effects of hyperinsulinemia on sex steroid availability [9,10] and IGF-1 production [11,12]. Hormone-independent mechanisms, including chronic inflammation with elevated levels of pro-inflammatory cytokines, infiltration of fat deposits by pro-inflammatory macrophages, and associated oxidative stress, have also been suggested [13].

Women with gestational diabetes mellitus (GDM) have an increased risk of diabetes, even in the first few years following delivery [14,15,16]. In addition, GDM shares underlying mechanisms with type 2 diabetes, including hyperglycemia due to pancreatic β-cell dysfunction coupled with insulin resistance and hyperinsulinemia. It is therefore possible that GDM may be associated with an increased risk of cancer [17,18,19,20,21,22,23,24,25], including BC.

Several studies have explored the association between GDM and cancer. This association was quantitatively analyzed in a meta-analysis by Wang et al. in 2020 [26]. This meta-analysis was not focused exclusively on BC and gave us few explanations for the discrepancies between the studies. Indeed, studies on this topic show divergent results. Wang et al. reported an odds ratio (OR) of 0.88 (95% CI 0.69; 1.12) for case-control studies and a relative risk (RR) of 1.08 (95% CI 0.89; 1.32) for cohort studies. They found three studies showing a statistically significant increased risk of BC after GDM, two studies showing a statistically significant protective effect, and ten studies showing non-significant results. Similarly, the most recent meta-analysis focusing exclusively on GDM and risk of BC found no significant association between GDM and BC in case-control studies (pooled OR = 0.85, 95% CI 0.65; 1.10) or in cohort studies (pooled RR = 1.00, 95% CI 0.80; 1.25) [27]. These studies demonstrate that the relationship between GDM and BC may be complex.

Therefore, the objective of this literature review was to compile the hypotheses studied and the methods employed in the literature that may explain this complex relationship and to encourage future studies on this subject.

## 2. Materials and Methods

We performed this review using the PubMed/Medline and Web Of Science databases using the following search terms: (gestational diabetes* OR gestational diabetes mellitus OR GDM) AND (breast cancer OR breast tumour OR breast neoplasm*), in accordance with the Preferred Reporting Items for Systematic Reviews and Meta-Analyses (PRISMA) guidelines (http://prisma-statement.org/prismastatement/Checklist.aspx 22 July 2021). The reference lists of previous reviews of the subject and of studies included in the analysis were also searched for any further relevant studies. All data extraction was performed by one researcher.

**Figure 1:**
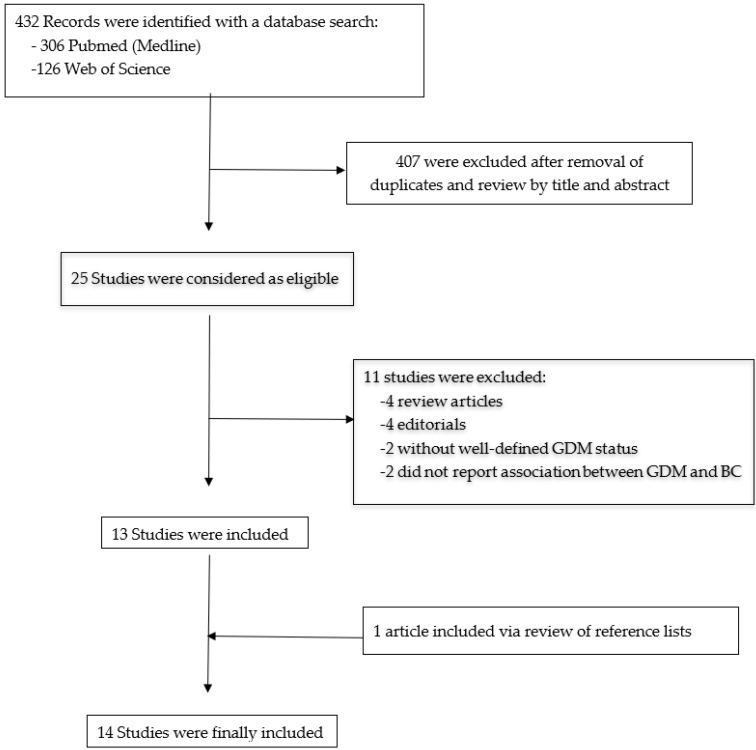
Flow diagram of the article selection process. GDM: gestational diabetes mellitus; BC: breast cancer.

Only English-language articles published before 1 June 2021, were included. We included all types of clinical studies reporting the association between GDM and BC with statistics available for adjusted odds ratio (OR) or relative risk (RR) and 95% confidence intervals (CI). Studies without original data, including reviews, comments, editorials, and meta-analyses were not included in the results but were used in the discussion to provide an additional perspective. Figure 1 shows the study selection process.

Data extracted included first author, publication year, country, specificities of the study population, sample size, duration of follow-up, criteria for GDM diagnosis, GDM rate, criteria for BC diagnosis, age or menopausal status at BC diagnosis, BC rate, adjusted variables, main results and sensitivity or additional analyses. These elements allowed us to qualitatively assess each study and to compare the results in view of the biases and strengths of each study. The risk of potential bias and the quality of each of the included studies was assessed using the Newcastle-Ottawa Scale (NOS), which is a standardized scale for assessing the bias and quality of non-randomized studies. (http://www.ohri.ca/programs/clinical_epidemiology/oxford.asp, 1 September 2021).

## 3. Results

### 3.1. Literature Search

A total of 732 articles were reviewed by title and abstract for this literature review. Among them, 25 were included for full-text review. Finally, 14 studies that met the inclusion criteria were included in the analysis—ten had a cohort design [19,20,21,22,23,24,28,29,30,31] and four had case-control designs [32,33,34,35]. One case-control study, Ardalan et al. [35], was added after a review of reference lists because it met the inclusion criteria; it investigated the relationship between gestational age and BC risk but also reported a measure of the association between GDM and BC.

### 3.2. Characteristics of Included Studies

Table 1 shows the detailed characteristics of the ten cohort studies and Table 2 shows the detailed characteristics of the four case-control studies. The studies are listed by year of publication in ascending order.

#### 3.2.1. Cohort Studies

Of the ten cohort studies, five were conducted in North America, three in Israel, and two in Asia. The first was published in 2008, and at least one study was published each year between 2015 and 2020. The cohorts were rather large, from 37,980 subjects for Perrin et al. (2008) [28] to 990,572 subjects for Peng et al. (2019) [23]. However, the vast majority of these studies recruited their populations before the most recent diagnostic criteria for GDM was published in 2010 [36]. The oldest cohorts were mostly prospective cohorts based on medical records and cancer registry data, while the most recent cohorts were mostly retrospective and based on self-reported or medico-administrative data.

It should also be noted that some studies had specific inclusion criteria, for example, Powe et al. [29] included only nurses, Park et al. [30] included only sisters of women with diagnosed BC, Han et al. [21] included only patients with a national screening examination within two years of pregnancy, and Bertrand et al. [31] included only African-American women. Follow-up was long in some studies, up to several decades in Perrin et al. [28] (median follow-up of 34 years), but in other studies, follow-up lasted only a few weeks for some of the cases (e.g., Bertrand et al. [31] and Fuchs et al. [22]). The rates of GDM and BC differed between these four studies, ranging from 1% to 9.4% and 0.3% to 5.4%, respectively. Only three cohort studies clearly stated if the BC was in situ or invasive [19,29,31], and two cohort studies differentiated between hormone-dependent and non-hormone-dependent cancers [29,31].

The results of these studies also likely differed due to the choice of adjustment variables. We found three studies showing a statistically significant increased risk of BC after GDM, two studies showing a statistically significant protective effect, and five studies showing non-significant results. Concerning the adjustment variables, it should be noted that Fuchs et al. [22]. published only univariate results and found a statistically significant protective effect [22]. However, Bejeimal et al. only took into account the number of visits to the doctor and found a statistically significant increased risk [24]. The vast majority of the other cohort studies adjusted for age, parity, ethnicity, or BMI. Four studies [19,20,28,30] included socio-economic variables (e.g., education, social class) and five studies [19,21,29,30,31] took into account lifestyle habits (smoking, alcohol, physical activity, contraception).

#### 3.2.2. Case-Control Studies

The four case-control studies were conducted in the US. They were published between 1998 and 2016 and included between 630 and 3174 subjects. These studies were based on self-reported data for the diagnosis of GDM, except for Ardalan et al. [35], which was based on recorded birth certificates. The diagnosis of BC was based on medical records, except for Rollison et al. [33], which was based on a cancer registry. Only one case-control study clearly presented whether BC was in situ or invasive [33] and two case-control studies specified hormone-dependent and non-hormone-dependent cancers [33,34].

In Troisi et al. [32] and Ardalan et al. [35], the subjects were younger than in the other two studies and the GDM rate ranged from 1.4% to 7.5%. It should also be noted that Rollison et al. [33] only included non-Hispanic whites, Hispanics, and American Indian women.

Among the case-control studies, we found only one study showing a statistically significant protective effect of GDM and three studies showing non-significant results. Regarding adjustment variables, all case-control studies included age and parity, three studies considered menopausal status and ethnicity, two took into account BMI, two took into account family history of BC, education, and alcohol consumption. Finally, one adjusted for physical activity and another for smoking habits.

### 3.3. Risk of Bias in the Included Studies

Table 3 and Table 4 show, respectively, the risk of bias for cohort studies and case-control studies, assessed via star rating according to the NOS.

Among the ten cohort studies, three studies had nine stars, three studies had eight stars, one study had seven stars, three studies had six stars, and another had five stars. Two studies selected the group of users (nurses, participants in a screening) [21,29]. In two studies, the diagnosis of GDM was self-reported [30,31]. In three studies, the outcome of interest was not present at the start [20,29,31]. In two studies, the follow-up was not long enough for outcomes to occur [22,31]. Five studies had a follow-up rate below 94% and/or no description of patients who were lost to follow-up [19,22,29,30,31]. In the study by Park et al. [30], the diagnosis of BC was self-reported and confirmed by medical records in only 81% of the cases [30].

Among the four case-control studies, three studies had six stars and one study had seven stars. Two studies did not have an adequate case definition, with record linkage using registers [33,35]. Three studies did not describe their source for the definition of controls and interviews were not blinded to case/control status [32,33,34]. In the study by Troisi et al. [32], the response rate to the interview was lower for controls than for cases [32]. In Ardalan et al. [35], there were no controls for GDM, given that the main objective of this study was to investigate the relationship between gestational age and BC [35].

## 4. Discussion

In this literature review, we found 14 studies investigating the relationship between GDM and BC risk. Among the 10 cohorts, three studies showed a statistically significant increased risk of BC after GDM, two studies showed a statistically significant protective effect, and five studies reported non-significant results. Among the four case-control studies, one study showed a statistically significant protective effect and three studies reported non-significant results. The 14 studies were of varying quality and used different methodologies. Using the NOS assessments, only 3 out of the 14 obtained all stars, which could explain the diversity of the results.

The case-control studies were all conducted in the US. The cohort studies showing either non-significant results or a statistically significant protective effect were also mostly carried out in North America. All the studies showing a statistically significant increased risk were carried out on the Asian continent (Israel, China). Regarding ethnicity and BC risk, Perrin et al. [28] showed that women of West Asian and North African origin have a significantly lower risk of developing BC. Therefore, a first hypothesis would be that ethnicity, culture, and/or country of residence has an influence on the relationship between GDM and BC.

A second hypothesis would be that the heterogeneity of the results may be partly explained by the use of different definitions of exposure (i.e., the diagnosis of GDM), which resulted in different rates of GDM. In the case-control studies, the history of GDM was self-reported, except for the study Ardalan et al. [35], which used birth certificate records, resulting in a GDM rate of 1.4%, but the exhaustiveness of the certificates is questionable. In the study by Troisi et al. [32], the women were young (between 20 and 44 years old), so, it can be assumed that the time between the diagnosis of GDM and the subjects’ responses was rather short, and they found a higher GDM rate of 7.5%. In contrast, the other two case-control studies had older populations, which may have led to recall bias, and the GDM rate was lower at around 3.3%. It should also be taken into account that the diagnostic criteria for GDM have evolved over the last 50 years, and earlier criteria detected only the most severe forms of GDM [36]. For example, the study by Perrin et al. [28] on pregnancies between 1964 and 1976 showed a GDM rate of 1%, whereas the more recent cohort studies report a rate of between 4.5% and 6%.

There is also the question of the treatment and monitoring of GDM. If the pathophysiological mechanism of an increased risk of BC is a combination of hyperglycemia coupled with insulin resistance, and hyperinsulinemia is presumed, then GDM balanced by regular follow-up and diet should not generate an excess risk compared with poorly followed or unbalanced GDM. Indeed, in 2004, Dawson et al. showed that “after adjusting for age, BMI, and smoking behavior, the risk of BC was found to be 1.2 to 5.1 times higher, respectively, in the highest quartile compared with the lowest quartile depending on the glucose indices used” [37]. One hypothesis would be that insulin-treated GDM may increase the risk of BC but not GDM balanced with diet.

We could go further by assuming that the protective effect of GDM found in several studies could be explained by the improvement of lifestyle habits (physical activity, diet) over time. However, in our literature review the one study that explored these elements (Powe et al. [29]) reported that GDM had a protective effect, but there was no significant differences in diet and physical activity between women with and without a history of GDM. However, it should also be noted that Powe et al. [29] focused on nurses, who are more likely to be conscious about their health, but who work at night, which is known as a BC risk factor [38].

According to the literature, some pathophysiological mechanisms could explain the protective effect, such as the altered hormonal milieu of a pregnancy complicated by GDM conferring protection to breast tissue. Pregnancy is known to induce proliferation and functional differentiation of breast lobules and ducts, which may explain the association between pregnancy history and BC risk [39,40,41]. Differences in circulating growth factors or hormones in GDM pregnancies may also have an impact on these processes and future BC risk.

Similarly, the criteria for diagnosing BC differ between studies and may lead to various classification biases. For instance, we can take the study by Park et al. [30] where the diagnosis of BC was self-reported and was confirmed by medical records in only 81% of cases. Cancer registries can also lead to a classification bias if they are not complete or up to date. The same applies to the completeness of medico-administrative databases based on hospital admissions, and the question arises for cancers that do not always require hospitalization, such as BC. Furthermore, only four studies clearly presented whether the cancer was in situ or invasive [19,29,31,33] and only four studies differentiated between hormone-dependent and non-hormone-dependent cancers [29,31,33,34], even though these cancers do not have the same pathophysiology and the same risk factors. Additionally, it can be assumed that patients in the control group may have had undetected BC, thus contributing to another classification bias.

The included studies also had very heterogeneous adjustment variables. Among the six cohort studies that did not focus solely on BC, most did not adjust for several known BC risk factors (e.g., oral contraceptive use, breastfeeding, alcohol consumption, or family history of BC). Only Powe et al. [29] and Bertrand et al. [31] adjusted for these risk factors, excluding alcohol consumption. Among the case-control studies, Troisi et al. [32] and Ardalan et al. [35] adjusted for alcohol consumption, while Rollison et al. [33] and Brasky et al. [34] adjusted for family history of BC; no case-control study adjusted for oral contraceptive use. There are also two risk factors for BC that are also known risk factors for GDM—obesity and physical inactivity. Therefore, it can be assumed that these factors play a confounding role in the relationship between BC and GDM [42], as well as type 2 diabetes. Thus, several studies have shown that GDM increased the risk of type 2 diabetes [14,16], and type 2 diabetes is recognized as increasing the risk of BC [43]. Although the vast majority of studies adjusted for BMI, only three studies adjusted for physical activity and none adjusted for type 2 diabetes in the main analyses. However, in their secondary analysis, Bejaimal et al. [24] showed that after adjustment for type 2 diabetes, the association between GDM and risk of BC remained significant (HR = 0.85, 95% CI [0.74; 0.99]). After a stratification on type 2 diabetes, Powe et al. [29] showed that GDM had a statistically significant protective effect on the risk of BC. In an additional analysis in their population of nurses, they showed that those with a history of GDM and type 2 diabetes had an even lower risk of BC (HR = 0.26, 95% CI [0.10; 0.68]).

Several studies have also shown that age is an important factor to take into account. Firstly, regarding age at onset of GDM, Rollison et al. [33], who showed a statistically significant protective effect of GDM on BC risk in their population, showed an even more protective effect in women who were younger than 35 years at onset of GDM (OR = 0.56, 95% CI [0.38; 0.82]). Secondly, the age at diagnosis of BC or the menopausal status of patients would influence the relationship between GDM and BC risk. For example, Perrin et al. [28] showed that there is a significantly increased risk with a history of GDM for BC diagnosed after age 50 (RR = 1.7 CI 95% [1.1; 2.5]).

Regarding BC subtypes, three studies adjusted or stratified on estrogen and/or progesterone receptor (ER/PR) status. No study worked on HER2 status. Bertrand et al. [31] did not show a statistically significant association between GDM and BC by estrogen receptor status. However, Rollison et al. [33] and Powe et al. [29], who had already found a statistically significant protective effect of GDM on BC risk, found an even more protective relationship for hormone-receptor-positive BC. However, this statistically significant relationship in Rollison et al. [33] was only found in women with GDM diagnosed before age 35. It would be interesting to further investigate this relationship between GDM and the different subtypes of BC. Only one study considered the effect of a history of multiple occurrences of GDM on the risk of BC. This study by Park et al. [30] was conducted in a particular population of sisters of women who had both BC and history of more than one occurrence of GDM. They studied the cumulative effect of the number of GDM occurrences and showed a statistically significant increased risk of BC.

Genomic characteristics were not discussed by any of the studies included in our literature review, even though genetic mutations could influence BC and GDM. We found several studies showing an increased frequency of certain mutations in BC patients. The most well-known germline mutations are BRCA1 and BRCA2, but others, such as PALB2 or TP53, may also be found [44,45,46,47]. Similarly, several hypotheses have been formulated regarding the link between genome mutations, such as GCK, HLA antigens, INSR, IGF2, HNF4A, INS-VNTR, HNF4a, CAPN10, MBL2, KCNJ11, ABCC8, ND1, TCF7L2, ADIPOQ, and PAI-1, and the development of GDM [48,49,50]. Chen et al. also suggested that mitochondrial DNA mutations could promote the development of GDM in some patients [51]. In addition, there are genetic mutations that could have an impact on the development of both GDM and BC, such as mutations in the ACE I/D gene [52,53]. The literature provides several hypotheses about the link between GDM, genetic mutations, and cancer risk. For example, Witczak et al. explained that: “gestational diabetes mellitus (GDM) is defined as carbohydrate intolerance resulting in hyperglycemia first diagnosed during pregnancy. It is associated with increased levels of oxidative stress due to overproduction of reactive oxygen species (ROS). The overproduction of ROS induces protein oxidation, lipid peroxidation and various types of DNA damage” [54]. However, it can be assumed that the currently established follow-up and treatments for GDM reduce the consequences on DNA. It would, therefore, be interesting in future studies to clarify the link between genotypes and the influence that they may have on the risk of GDM and/or BC in order to improve prevention and treatment. Regarding the length of follow-up required to study the relationship between GDM and BC risk, the existing literature has not clearly established the ideal length. If the follow-up is less than 15 years, as in the vast majority of cohorts in this review, there is a risk of studying predominantly pre-menopausal BC. A follow-up of more than 15 years would make it possible to evaluate the effects of GDM in the longer term, particularly on menopausal BC. While studies suggest that type 2 diabetes and obesity are not association with premenopausal BC or with a lower risk [19,28,55], high BMI is positively associated with risk of postmenopausal BC [56]. A large sample size is also required in this type of study since the BC rate is usually less than 1%.

Our literature review has several limitations. Firstly, it was conducted by a single reviewer. However, we were able to rely on two recent meta-analyses [26,27] to ensure that our review was exhaustive. Secondly, it was difficult to make a straightforward comparison between studies. Indeed, the study populations were either too small, with respect to the number of cases, to have sufficient power, or too unrepresentative (majority of studies done in the US, with a particular ethnicity, with cohorts of nurses, based on participation in a national health screening examination, or with a cohort of sisters of women with BC). There were also potential selection biases, including incomplete follow-up in cohort studies and low response rates in case-control studies. Exposure misclassification is another particular methodological issue and several studies included in this systematic review used self-reported estimates instead of objective clinical diagnosis based on biochemical testing [57]. The adjustment variables are also very heterogeneous and sometimes do not correspond to known risk factors for GDM or BC.

## 5. Conclusions

To conclude, the results of studies addressing the link between GDM and BC seem to be inconsistent and they should be interpreted with caution seeing as many present quantitative or qualitative methodological issues. The link between GDM and BC, therefore, warrants further investigation and a better understanding of the associations and the underlying mechanisms is needed. It would be advisable for studies to be conducted not only in North America and Asia, but also in the rest of the world, and for them to be representative of their populations. They should have a clear and up-to-date definition of GDM, as well as the different types of BC (menopausal or not, hormone-dependent or not, invasive or not). It would be useful to know what measures or treatments were put in place after the diagnosis of GDM and to study the cumulative effect of GDM. It would also be interesting to adjust for known risk factors for BC and GDM, especially obesity and/or physical inactivity, as well as type 2 diabetes, which could be considered as potential confounders in this relationship.

## Figures and Tables

**Table 1:** Characteristics of the cohort studies.

First Author	Publication Year	Country	Specificities of the Study Population	Sample Size	Duration of Follow-Up	Criteria for GDM Diagnosis	GDM Rate	Criteria for BC Diagnosis	BC Rate	Adjusted Variables	RR [CI 95%]	Sensitivity or Additional Analyses
Perrin [28]	2007	Israel	Deliveries between 1964 to 1976	37,980	Until 2015	Medical records	1%	Cancer registry	4.3%	Age, parity, social class, ethnic origin, education, immigration status	1.5 [1.0; 2.1] P = 0.03	Age at diagnosis of BC: <50 years old RR = 1 [0.5; 2.1] ≥50 years old RR = 1.7 [1.1; 2.5]
Sella [19]	2011	Israel	Aged between 15–50 years Screened between 13 March 1995 and 27 May 2009	185,315	Until June 1st 2009 (mean 5 years)	Medical records	6%	Cancer registry	0.3%	Age, socioeconomic level, smoking, BMI, gravidity, number of general practitioner visits	0.87 [0.63; 1.20]	-
Bejaimal [24]	2016	Canada	Aged between 20–50 years Screened between 1 January 1995 and July 4th 2008	149,049: 49,684 women with GDM matched on age and year of delivery in a ratio of 1:2 to pregnant women without GDM	Until 31 December 2011	Medical Records	4.6%	Cancer Registry	-	Number of physician visits	0.86 [0.75; 0.98]	After adjustment for future diabetes, the association between GDM and risk of breast cancer remained significant (HR 0.85 [0.74; 0.99])
Fuchs [22]	2017	Israel	Deliveries between 1988 to 2013	104,715	Until 2013	Medical records	9.4%	Medical records	0.5%	Univariate results only	1.8 [1.5; 2.1]	/
Powe [29]	2017	US	Only nurses who declared a delivery at the beginning of the cohort in 1989 or with incident delivery through 2001	86,972	Until 2013	Self-reported, 94% confirmed by medical records	6%	Medical records	2.7%	Age, BMI at 18 years old, weight gain since age 18, height, physical activity, alcohol intake, age at menarche, birth index, breastfeeding, hormone therapy use, history of breast cancer in mother or sister, history of benign breast disease, ethnicity, mammography within the past 2 years	0.72 [0.58; 0.89]	T2D only: HR = 0.69 [0.40; 1.18] GDM + T2D: HR = 0.26 [0.10; 0.68] GDM and hormone receptor positive BC: HR 0.65 [0.50–0.84], GDM and hormone receptor negative BC: HR 0.96 [0.60–1.52]
Park [30]	2017	US	35–74 years old, all breast-cancer-free and sisters of women with BC between 2003 and 2009	39,198	Until 14 August 2015 (mean: 7.4 years)	Self-reported	4.2% (0.9% multiple GDM pregnancies)	Self-reported, 81% confirmed by medical records	5.4%	Age, ethnicity, education, age at first birth, age at menarche, weight at age 10, BMI at 30–39 years old, physical activity in their childhood and teens	1.10 [0.88; 1.36]	1 GDM pregnancy: HR = 0.94 [0.73; 1.22], more than 1 GDM pregnancies HR = 1.68 [1.15; 2.44]
Han [21]	2018	South Korea	1st pregnancy between 2004 and 2005 and participated in a “National Health Screening examination” at least 2 years before their first delivery	102,900	Until 2015	Medico-administrative database ICD 10 codes O 24.4 and O 24.9	4.83%	Medico-administrative database ICD 10 codes	0.7%	Age, smoking, BMI before pregnancy	1.15 [0.83; 1.58]	-
Peng [23]	2019	Taiwan	All deliveries between 2002 and 2012	990,572	Until 31 December 2013	Medico-administrative database ICD 9 codes 648-250	4.8%	Medico-administrative database ICD 9 codes	0.47%	Age, hypertension, dyslipidemia, liver disease, infertility and kidney disease	1.23 [1.09; 1.39]	-
Pace [20]	2020	Canada	34,294 randomly selected deliveries between April 1990 and 31 December 2007 with 2 or more diagnostic codes for GDM were matched 1:1 to 34,294 deliveries without GDM (by year of delivery, age, region)	68,588	Until 2012 (mean: 13.1 years)	2 or more GDM codes	-	Hospital discharge abstracts using ICD codes	1.1%	Gestational hypertension, preterm delivery, infant size, parity, prior comorbidity, material deprivation index, ethnicity	0.93 [0.80; 1.09]	-
Bertrand [31]	2020	US	African American parous women between March 1995 and March 2017	41,767	Until March 2017	Self-reported	4.9%	Self-reported and confirmed in cancer registry records or medical records	4%	Age, BMI at age 18, recent BMI, parity, menarche, age at first birth, oral contraceptive duration, and family history of BC	0.98 [0.77; 1.25]	No significant results after adjustment on ER status

GDM: gestational diabetes mellitus; BC: breast cancer; RR: relative risk; HR: hazard ratio; BMI: body mass index; T2D: type 2 diabetes; ICD: International Classification of Disease.

**Table 2:** Characteristics of the case-control studies.

First Author	Publication Year	Country	Specificities of the Study Population	Sample Size	Criteria for GDM Diagnosis	GDM Rate	Criteria for BC Diagnosis	Adjusted Variables	OR [CI 95%]	Sensitivity or Additional Analyses
Troisi [32]	1998	US	Age between 20 and 44 years	3174	Self-report	7.5%	Medical records	Age at menarche, mammography, alcohol, BMI, site, race, combination variables representing parity and age at first birth	1.1 [0.7; 1.4]	-
Rollison [33]	2008	US	Non-Hispanic white, Hispanic, and American Indian women (mean age: 56 years)	2523	Self-report	3.2%	Cancer registry	Age, study site, menopausal status, BMI, BMI at 15 years old, parity, age at menarche, physical activity, family history of BC, breastfeeding history	0.71 [0.52; 0.98]	Stratification on age at onset of GDM: 0.56 [0.38; 0.82] ≥ 35 years old 1.34 [0.72; 2.52] ≥ 35 years old Stratification on age at onset of GDM and ER/PR status: <35 years old and ER + 0.52 [0.31; 0.85] <35 years old and PR + 0.53 [0.32; 0.89] ≥35 years old and ER − 2.52 [1.07; 5.53] ≥35 years old and PR − 3.08 [1.45; 6.54]
Brasky [34]	2013	US	Age between 35 and 79 years	2812	Self-report	3.3%	Medical records	Age, education, history of benign breast disease, family history of BC, age at first pregnancy, number of pregnancies, menopausal status, age at menopause	0.79 [0.48; 1.30]	-
Ardalan [35]	2016	US	Women diagnosed with premenopausal BC (age 21–49) within the 5 years after delivering a live baby between 1 January 1994 and 31 December 2003	630	Recorded birth certificates	1.4%	Cancer registry	Mother’s age at delivery, race/ethnicity, level of education, birth weight, parity, gestational age, weight gain in pregnancy, smoking habits, drinking habits, induction of labor, gestational hypertension	1.62 [0.30; 8.68]	-

GDM: gestational diabetes mellitus; BC: breast cancer; OR: odds ratio; BMI: body mass index; ER, estrogen receptor; PR, progesterone receptor.

**Table 3:** Risk of bias and quality assessment of cohort studies.

		Selection				Comparability		Outcome			
First Author	Publication Year	Representativeness of the Exposed Cohort	Selection of the Non-Exposed Cohort	Ascertainment of Exposure	Demonstration that Outcome of Interest Was Not Present at Start of Study	Controls for the Most Important Factor	Controls for any Additional Factor	Assessment of Outcome	Was Follow up Long Enough for Outcome to Occur	Adequacy of Follow up of Cohorts	Total
Perrin [28]	2008	*	*	*	*	*	*	*	*	*	9
Sella [19]	2011	*	*	*	*	*	*	*	*		8
Bejaimal [24]	2015	*	*	*	*	*	*	*	*	*	9
Fuchs [22]	2016	*	*	*	*	*	*	*			7
Powe [29]	2017		*	*		*	*	*	*		6
Park [30]	2017	*	*		*	*	*		*		6
Han [21]	2018		*	*	*	*	*	*	*	*	8
Peng [23]	2019	*	*	*	*	*	*	*	*	*	9
Pace [20]	2020	*	*	*		*	*	*	*	*	8
Bertrand [31]	2020	*	*			*	*	*			5

Note: Assessments are based on Newcastle–Ottawa Scale. ‘high’ quality choices are identified with a ‘*’.

**Table 4:** Risk of bias and quality assessment of case-control studies.

		Selection				Comparability		Exposure			
First Author	Publication Year	Is the Case Definition Adequate?	Representativeness of the Cases	Selection of Controls	Definition of Controls	Controls for the Most Important Factor	Controls for Any Additional Factor	Ascertainment of Exposure	Same method of Ascertainment for Cases and Controls	Non-Response Rate	Total
Troisi [32]	1998	*	*	*		*	*		*		6
Rollison [33]	2008		*	*		*	*		*	*	6
Brasky [34]	2013	*	*	*		*	*		*	*	7
Ardalan [35]	2016		*	*	*			*	*	*	6

Note: Assessments are based on Newcastle–Ottawa Scale. ‘high’ quality choices are identified with a ‘*’.

## Data Availability

We performed this review using the PubMed/Medline and Web Of Science databases using the following search terms: (gestational diabetes* OR gestational diabetes mellitus OR GDM) AND (breast cancer OR breast tumour OR breast neoplasm*), in accordance with the Preferred Reporting Items for Systematic Reviews and Meta-Analyses (PRISMA) guidelines.

## References

[1] Sung H., Ferlay J., Siegel R.L., Laversanne M., Soerjomataram I., Jemal A., Bray F. (2021). Global Cancer Statistics 2020: GLOBOCAN Estimates of Incidence and Mortality Worldwide for 36 Cancers in 185 Countries. CA A Cancer J. Clin..

[2] Michels K.B., Solomon C.G., Hu F.B., Rosner B.A., Hankinson S.E., Colditz G., Manson J.E. (2003). Type 2 Diabetes and Subsequent Incidence of Breast Cancer in the Nurses’ Health Study. Diabetes Care.

[3] Xue F., Michels K.B. (2007). Diabetes, metabolic syndrome, and breast cancer: A review of the current evidence. Am. J. Clin. Nutr..

[4] Larsson S.C., Mantzoros C.S., Wolk A. (2007). Diabetes mellitus and risk of breast cancer: A meta-analysis. Int. J. Cancer.

[5] Liao S., Li J., Wei W., Wang L., Zhang Y., Li J., Wang C., Sun S. (2011). Association between diabetes mellitus and breast cancer risk: A meta-analysis of the literature. Asian Pac. J. Cancer Prev..

[6] Boyle P., Boniol M., Koechlin A., Robertson C., Valentini F., Coppens K., Fairley L.-L., Zheng T., Zhang Y., Pasterk M. (2012). Diabetes and breast cancer risk: A meta-analysis. Br. J. Cancer.

[7] Linde J.S., Karlstad O., Eriksen S.A., Vestergaard P., Bronsveld H.K., de Vries F., Andersen M., Auvinen A., Haukka J., Hjellvik V. (2013). CARING (CAncer Risk and INsulin analoGues): The Association of Diabetes Mellitus and Cancer Risk with Focus on Possible Determinants-A Systematic Review and a Meta-Analysis. Curr. Drug Saf..

[8] Dankner R., Boffetta P., Balicer R.D., Boker L.K., Sadeh M., Berlin A., Olmer L., Goldfracht M., Freedman L.S. (2016). Time-Dependent Risk of Cancer After a Diabetes Diagnosis in a Cohort of 2.3 Million Adults. Am. J. Epidemiol..

[9] Nyholm H., Djursing H., Hagen C., Agner T., Bennett P., Svenstrup B. (1989). Androgens and Estrogens in Postmenopausal Insulin-Treated Diabetic Women. J. Clin. Endocrinol. Metab..

[10] Lipworth L., Adami H.-O., Trichopoulos D., Cartström K., Mantzoros C. (1996). Serum Steroid Hormone Levels, Sex Hormone-Binding Globulin, and Body Mass Index in the Etiology of Postmenopausal Breast Cancer. Epidemiology.

[11] Lawlor D.A., Smith G.D., Ebrahim S. (2004). Hyperinsulinaemia and increased risk of breast cancer: Findings from the British Women’s Heart and Health Study. Cancer Causes Control..

[12] Chaudhuri P.K., Chaudhuri B., Patel N. (1986). Modulation of estrogen receptor by insulin and its biologic significance. Arch. Surg..

[13] Ferroni P., Riondino S., Buonomo O., Palmirotta R., Guadagni F., Roselli M. (2015). Type 2 Diabetes and Breast Cancer: The Interplay between Impaired Glucose Metabolism and Oxidant Stress. Oxidative Med. Cell. Longev..

[14] Bellamy L., Casas J.-P., Hingorani A., Williams D. (2009). Type 2 diabetes mellitus after gestational diabetes: A systematic review and meta-analysis. Lancet.

[15] Kaul P., Savu A., Nerenberg K.A., Donovan L.E., Chik C.L., Ryan E.A., Johnson J. (2015). Impact of gestational diabetes mellitus and high maternal weight on the development of diabetes, hypertension and cardiovascular disease: A population-level analysis. Diabet. Med..

[16] Retnakaran R., Shah B.R. (2016). Role of Type 2 Diabetes in Determining Retinal, Renal, and Cardiovascular Outcomes in Women With Previous Gestational Diabetes Mellitus. Diabetes Care.

[17] Wartko P.D., Beck T.L., Reed S., Mueller B.A., Hawes S.E. (2017). Association of endometrial hyperplasia and cancer with a history of gestational diabetes. Cancer Causes Control.

[18] Perrin M.C., Terry M.B., Kleinhaus K., Deutsch L., Yanetz R., Tiram E., Calderon R., Friedlander Y., Paltiel O., Harlap S. (2007). Gestational diabetes as a risk factor for pancreatic cancer: A prospective cohort study. BMC Med..

[19] Sella T., Chodick G., Barchana M., Heymann A.D., Porath A., Kokia E., Shalev V. (2011). Gestational diabetes and risk of incident primary cancer: A large historical cohort study in Israel. Cancer Causes Control.

[20] Pace R., Rahme E., Dasgupta K. (2020). Gestational diabetes mellitus and risk of incident primary cancer: A population-based retrospective cohort study. J. Diabetes.

[21] Han K.-T., Cho G.J., Kim E.H. (2018). Evaluation of the Association between Gestational Diabetes Mellitus at First Pregnancy and Cancer within 10 Years Postpartum Using National Health Insurance Data in South Korea. Int. J. Environ. Res. Public Health.

[22] Fuchs O., Sheiner E., Meirovitz M., Davidson E., Sergienko R., Kessous R. (2017). The association between a history of gestational diabetes mellitus and future risk for female malignancies. Arch. Gynecol. Obstet..

[23] Peng Y.-S., Lin J.-R., Cheng B.-H., Ho C., Lin Y.-H., Shen C.-H., Tsai M.-H. (2019). Incidence and relative risk for developing cancers in women with gestational diabetes mellitus: A nationwide cohort study in Taiwan. BMJ Open.

[24] Bejaimal S.A.D., Wu C.F., Lowe J., Feig D.S., Shah B.R., Lipscombe L.L. (2016). Short-term risk of cancer among women with previous gestational diabetes: A population-based study. Diabet. Med..

[25] Simon J., Goueslard K., Arveux P., Bechraoui-Quantin S., Petit J.-M., Quantin C. (2021). Increased Risk of Hospitalization for Pancreatic Cancer in the First 8 Years after a Gestational Diabetes Mellitus regardless of Subsequent Type 2 Diabetes: A Nationwide Population-Based Study. Cancers.

[26] Wang Y., Yan P., Fu T., Yuan J., Yang G., Liu Y., Zhang Z.-J. (2020). The association between gestational diabetes mellitus and cancer in women: A systematic review and meta-analysis of observational studies. Diabetes Metab..

[27] Xie C., Wang W., Li X., Shao N., Li W. (2017). Gestational diabetes mellitus and maternal breast cancer risk: A meta-analysis of the literature. J. Matern. -Fetal Neonatal Med..

[28] Perrin M.C., Terry M.B., Kleinhaus K., Deutsch L., Yanetz R., Tiram E., Calderon-Margalit R., Friedlander Y., Paltiel O., Harlap S. (2007). Gestational diabetes and the risk of breast cancer among women in the Jerusalem Perinatal Study. Breast Cancer Res. Treat..

[29] Powe C.E., Tobias D.K., Michels K.B., Chen W.Y., Eliassen A.H., Manson J.E., Rosner B., Willett W.C., Hu F.B., Zhang C. (2017). History of Gestational Diabetes Mellitus and Risk of Incident Invasive Breast Cancer among Parous Women in the Nurses’ Health Study II Prospective Cohort. Cancer Epidemiol. Prev. Biomark..

[30] Park Y.-M., O’Brien K.M., Zhao S., Weinberg C.R., Baird D.D., Sandler D.P. (2017). Gestational diabetes mellitus may be associated with increased risk of breast cancer. Br. J. Cancer.

[31] Bertrand K.A., Castro-Webb N., Cozier Y.C., Li S., O’Brien K.M., Rosenberg L., Palmer J.R. (2020). Gestational Diabetes and Risk of Breast Cancer in African American Women. Cancer Epidemiol. Prev. Biomark..

[32] Troisi R., Weiss H.A., Hoover R.N., Potischman N., Swanson C.A., Brogan D.R., Coates R.J., Gammon M.D., Malone K.E., Daling J.R. (1998). Pregnancy Characteristics and Maternal Risk of Breast Cancer. Epidemiology.

[33] Rollison D.E., Giuliano A.R., Sellers T.A., Laronga C., Sweeney C., Risendal B., Baumgartner K.B., Byers T., Slattery M.L. (2007). Population-based Case-Control Study of Diabetes and Breast Cancer Risk in Hispanic and Non-Hispanic White Women Living in US Southwestern States. Am. J. Epidemiol..

[34] Brasky T.M., Li Y., Jaworowicz D.J., Potischman N., Ambrosone C.B., Hutson A.D., Nie J., Shields P.G., Trevisan M., Rudra C.B. (2013). Pregnancy-related characteristics and breast cancer risk. Cancer Causes Control.

[35] Ardalan A., Bungum T. (2016). Gestational Age and the Risk of Maternal Breast Cancer: A Population-Based Case-Control Study. Breast J..

[36] Karagiannis T., Bekiari E., Manolopoulos K., Paletas K., Tsapas A. (2010). Gestational diabetes mellitus: Why screen and how to diagnose. Hippokratia.

[37] Dawson S.I. (2003). Long-term risk of malignant neoplasm associated with gestational glucose intolerance. Cancer.

[38] Cordina-Duverger E., Menegaux F., Popa A., Rabstein S., Harth V., Pesch B., Brüning T., Fritschi L., Glass D., Heyworth J. (2018). Night shift work and breast cancer: A pooled analysis of population-based case–control studies with complete work history. Eur. J. Epidemiol..

[39] Schedin P. (2006). Pregnancy-associated breast cancer and metastasis. Nat. Rev. Cancer.

[40] Russo I.H., Russo J. (2011). Pregnancy-Induced Changes in Breast Cancer Risk. J. Mammary Gland. Biol. Neoplasia.

[41] Meier-Abt F., Bentires-Alj M., Rochlitz C. (2015). Breast Cancer Prevention: Lessons to be Learned from Mechanisms of Early Pregnancy–Mediated Breast Cancer Protection. Cancer Res..

[42] Tankó L.B., Bagger Y.Z., Christiansen C. (2004). Long-term risk of malignant neoplasm associated with gestational glucose intolerance. Cancer.

[43] Chodick G., Zucker I. (2011). Diabetes, Gestational Diabetes and the Risk of Cancer in Women: Epidemiologic Evidence and Possible Biologic Mechanisms. Women’s Health.

[44] Tariq H., Gul A., Zubair M., Jaffer S.R., Zafar N., Sadaf G. (2021). Targeted Next-generation Sequencing for Reliable Detection of Genetic Status in Breast Cancer. J. Coll. Physicians Surg. -Pak. JCPSP.

[45] Guglielmi C., Scarpitta R., Gambino G., Conti E., Bellè F., Tancredi M., Cervelli T., Falaschi E., Cosini C., Aretini P. (2021). Detection of Germline Variants in 450 Breast/Ovarian Cancer Families with a Multi-Gene Panel Including Coding and Regulatory Regions. Int. J. Mol. Sci..

[46] Ren M., Orozco A., Shao K., Albanez A., Ortiz J., Cao B., Wang L., Barreda L., Alvarez C.S., Garland L. (2021). Germline variants in hereditary breast cancer genes are associated with early age at diagnosis and family history in Guatemalan breast cancer. Breast Cancer Res. Treat..

[47] Rogoża-Janiszewska E., Malińska K., Górski B., Scott R.J., Cybulski C., Kluźniak W., Lener M., Jakubowska A., Gronwald J., Huzarski T. (2021). Prevalence of germline TP53 variants among early-onset breast cancer patients from Polish population. Breast Cancer.

[48] Wise A., Nguyen T., Herring A., North K.E., Siega-Riz A.M., Stuebe A.M. (2013). Maternal Genotype and Gestational Diabetes. Am. J. Perinatol..

[49] Lambrinoudaki I., Vlachou S.A., Creatsas G. (2010). Genetics in gestational diabetes mellitus: Association with incidence, severity, pregnancy outcome and response to treatment. Curr. Diabetes Rev..

[50] Robitaille J., Grant A.M. (2008). The genetics of gestational diabetes mellitus: Evidence for relationship with type 2 diabetes mellitus. Genet. Med..

[51] Chen Y., Liao W., Roy A., Loganath A., Ng S. (2000). Mitochondrial gene mutations in gestational diabetes mellitus. Diabetes Res. Clin. Pract..

[52] Khan I.A., Jahan P., Hasan Q., Rao P. (2014). Angiotensin-converting enzyme gene insertion/deletion polymorphism studies in Asian Indian pregnant women biochemically identifies gestational diabetes mellitus. J. Renin-Angiotensin-Aldosterone Syst..

[53] Singh A., Srivastava N., Amit S., Prasad S., Misra M., Ateeq B. (2018). Association of AGTR1 (A1166C) and ACE (I/D) Polymorphisms with Breast Cancer Risk in North Indian Population. Transl. Oncol..

[54] Witczak M., Wilczyński J., Gulczyńska E., Talar T., Mordalska A., Łopaczyńska D., Ferenc T. (2017). What is the impact of gestational diabetes mellitus on frequency of structural chromosome aberrations in pregnant women and their offspring?. Mutat. Res. Genet. Toxicol. Environ. Mutagenesis.

[55] Cnattingius S., Torrång A., Ekbom A., Granath F., Petersson G., Lambe M. (2005). Pregnancy characteristics and maternal risk of breast cancer. Jama.

[56] Van den Brandt P.A., Spiegelman D., Yaun S.-S., Adami H.-O., Beeson L., Folsom A.R., Fraser G.R., Goldbohm A., Graham S., Kushi L. (2000). Pooled analysis of prospective cohort studies on height, weight, and breast cancer risk. Am. J. Epidemiol..

[57] Xie S., Yang S.-J., Chen J., Cheng J.-Q. (2015). Gestational Diabetes Mellitus and Subsequent Cancer Risk: Shared Risk Factors, Causality or Confounding?. Asian Pac. J. Cancer Prev..

